# Editorial: Role of RNA Modification in Disease

**DOI:** 10.3389/fgene.2019.00920

**Published:** 2019-10-23

**Authors:** Emanuele Buratti, Amit Bhardwaj

**Affiliations:** ^1^Molecular Pathology, International Centre for Genetic Engineering and Biotechnology (ICGEB), Trieste, Italy; ^2^Kimmel Center for Biology and Medicine at the Skirball Institute, Department of Pathology, New York University School of Medicine, New York, NY, United States

**Keywords:** RNA, RNA Splicing, RNA binding proteins, RNA modifications, Human disease and tRNA modifications

When first discovered, mRNA molecules were considered as the simple way through which the information stored in the DNA could be transformed in the real effector molecules, the cellular proteins. This view did not last very long because it became clear very soon that RNA molecules had the ability to determine the way the information was transferred from DNA to the proteins.

Today, it is very clear that RNA molecules play a fundamental role in two of the most complex macromolecular machineries found in cells: the spliceosome and the ribosome. Furthermore, the presence in our cells of a vast array of small and large noncoding RNAs (ncRNAs) whose function and regulation are still partially unknown has demonstrated that RNA can actively affect regulatory networks. In particular, the developmental and tissue-specific expression of these ncRNAs can profoundly affect constitutive and alternative mRNA regulatory processes and also participates in the fine tuning of translational processes. As a result of all this complexity, the emerging view is one where RNA molecules occupy a central position in almost all cellular processes within the cell, and only their correct expression can ensure both proper functioning and survival. Unfortunately, the presence of all these very complex regulatory networks also implicates that defects at the level of pre-RNA processing pathways are a major cause of human disease.

The purpose of this Research Topic has been to provide an overview of several issues that relate to this issue, with a special focus on new emerging aspects of disease-related RNA connections that range from studying modifications of the RNA molecule itself to the effect of proteins that regulate its processing.

Regarding the RNA molecule itself, the analysis of RNA *N* ^6^-methyladenosine (m^6^A) modifications has been recently suggested to play a critical role in a variety of biological processes and to be especially associated with cancer risk. In their approach, Tang et al. have developed an online database (DRUM) to support the query of disease-associated RNA m^6^A methylation sites that will help unravel disease mechanisms at the epitranscriptome layer.

Another important regulatory layer of RNA processing is represented by the many RNA binding proteins that are expressed within the eukaryotic nucleus. In particular, an increasingly important role is being played by classical hnRNP binding properties, as described by Silva et al. In their work, Silva et al. have investigated the importance of TDP-43 in frontotemporal dementia and that mice conditionally expressing this protein recapitulate several core behavioral features of Frontotemporal Dementia/Amyotrophic Lateral Sclerosis (FTD/ALS) spectrum of human pathology. This particular study highlights the importance of RNA binding proteins in neurodegenerative diseases Interestingly, many of these proteins have not yet been characterized very well, despite being known since a long time, and this particular topic has been analyzed by Cappelli et al. with regard to the genes controlled by the two closely related hnRNP Q and R in neuronal cell lines and that were previously shown to affect TDP-43 toxicity in flies and neuronal cell lines (Appocher et al., 2017).

Finally, much of the excitement that is taking place nowadays with regard to RNA metabolism is targeted at the use of small effectors to recover RNA alterations in a variety of diseases. Therefore, the two final chapters in this research topic look at RNA-based therapeutic strategies both in general terms (Harries) and in targeting a more specialized behavior of RNA binding proteins that is represented by their ability to undergo phase transitions (Verdile et al.).

## Author Contributions

Both authors contributed to the writing of this editorial.

## Conflict of Interest

The authors declare that the research was conducted in the absence of any commercial or financial relationships that could be construed as a potential conflict of interest.
